# Prognostic Value of Angiography-Derived Index of Microcirculatory Resistance in Patients with Coronary Artery Disease Undergoing Rotational Atherectomy

**DOI:** 10.31083/j.rcm2405131

**Published:** 2023-04-27

**Authors:** Bo Wang, Yue Gao, Yifan Zhao, Peng Jia, Jun Han, Hailing Li, Yi Zhang, Yawei Xu

**Affiliations:** ^1^Department of Cardiology, Shanghai Tenth People’s Hospital, Tongji University School of Medicine, 200072 Shanghai, China; ^2^Department of Cardiology, North Station Hospital of Jing’an District, 200072 Shanghai, China

**Keywords:** coronary angiography, index of microcirculatory resistance, coronary artery disease, coronary microvascular dysfunction, rotational atherectomy

## Abstract

**Background:**

Rotational atherectomy (RA) is the major tool used to treat severely calcified lesions in patients with coronary artery disease (CAD). The relationship between coronary microvascular dysfunction and RA remains unknown. Therefore, we attempted to explore the predictive implications of the coronary angiography-derived index of microcirculatory resistance (angio-IMR) in CAD patients undergoing RA.

**Methods:**

This retrospective study included 118 patients with severe coronary calcification who underwent a successful RA from January 2018 to June 2021. The angio-IMR was calculated based on computed flow and pressure dynamic principles to assess coronary microcirculatory function. Follow-up was performed on all patients for major adverse cardiovascular events (MACEs), including all-cause death, non-fatal myocardial infarction, target vessel revascularization (TVR), and stroke.

**Results:**

The mean angio-IMR for all patients was 25.58 ± 7.93. Patients were stratified the groups based on a mean angio-IMR of 25, fifty-four (45.8%) patients had angio-IMR ≥25. The logistic regression analysis showed that angiography-derived fractional flow reserve was significantly associated with coronary microvascular dysfunction. After median follow-up of 21.7 (15.1–24.0) months, MACEs occurred in 30.6%, including 12.5% all-cause deaths, 6.4% non-fatal myocardial infarction, 14.5% TVR, and 0.9% stroke. Kaplan-Meier analysis demonstrated that patients with angio-IMR ≥25 had greater cumulative MACEs (41.6%) and TVR (20.7%) than patients with preserved angio-IMR. COX regression analysis indicated that angio-IMR ≥25 and reduced left ventricular ejection fraction were independent predictors of MACEs. In addition, angio-IMR ≥25 and lowered minimum luminal area independently predicted TVR occurrence.

**Conclusions:**

In CAD patients undergoing RA, angio-IMR ≥25 was an independent and significant predictor of MACEs and TVR.

**Clinical Trial Registration:**

NCT05435898.

## 1. Introduction

Severe coronary artery calcification affects the outcome of percutaneous coronary interventions (PCI), impairs stent expansion and causes dilatation balloon rupture, increases the rate of stent target vessel failure and in-stent restenosis, and increases cardiac death [1]. Rotational atherectomy (RA) is a technique used to treat calcified lesions in coronary arteries, which can increase the rate of procedural success and improve patient prognosis, especially with the use of drug-eluting stents (DES). RA for plaque modification has become an important technique to improve the outcomes following PCI [2, 3]. The principle of RA is to use a rotating diamond-covered burr to differentially crush the stiff plaque and fibrous tissue into particles of 7.0–7.5 μm, the size which corresponds to the volume of red blood cells and therefore can be absorbed by the distal reticuloendothelium with the washout of blood flow [3, 4].

The coronary microcirculatory system is mainly composed of pre-arterioles (<500 μm) and arterioles (<200 μm) of the distal coronary arteries [5] Coronary microcirculatory dysfunction (CMD) is widely present in various coronary artery diseases (CADs), especially in ischemic non-obstructive CAD [6, 7, 8]. Previous studies suggest that CMD is associated with coronary microvascular spasm, endothelial disorders, and coronary microthrombotic obstruction [9]. The index of microcirculatory resistance (IMR) is currently the primary means of assessing microcirculatory function. Several studies have shown that angiography-derived IMR (angio-IMR) is in good agreement with wire-derived IMR and greatly simplifies the measurement process [10, 11].

However, it is unknown as to whether the formation of microparticles in severely calcified coronary lesions following RA increases the incidence of CMD, and the prognostic significance of CMD after RA. Consequently, in this study, we attempted to apply angio-IMR to assess coronary microvascular function in patients undergoing RA and follow these patients to assess their clinical outcomes.

## 2. Methods

### 2.1 Study Design and Patient Population

**Fig. 1.**
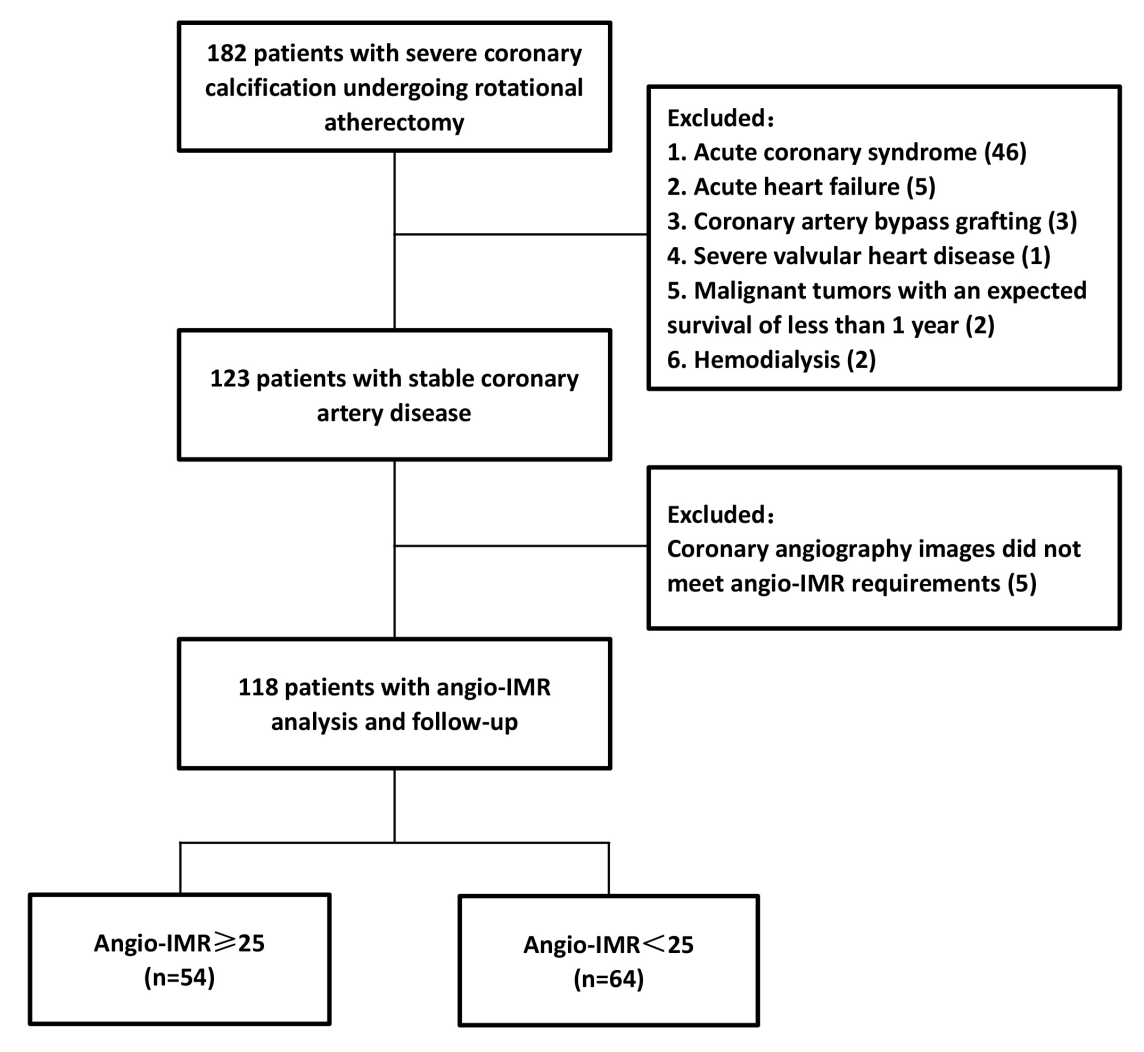
**Study flow chart**. Abbreviations: Angio-IMR, coronary angiography-derived index of microcirculatory resistance.

This is a retrospective, observational study to determine coronary microcirculation and follow-up outcomes in 118 patients with severe coronary calcified lesions following RA at the Department of Cardiology, Shanghai Tenth People’s Hospital, Shanghai, China, between January 2018 and July 2021. All patient demographics, co-morbidities, examination results and angiographic characteristics, and key procedural details were recorded through the medical record system. Patients with acute coronary syndromes (including acute myocardial infarction and unstable angina), coronary artery bypass grafting, severe valvular heart disease, acute heart failure, malignant tumors with an expected survival of less than one year, and hemodialysis patients were excluded. The flow chart of the study is shown in Fig. 1. All procedures performed on patients followed the Helsinki Declaration. The study protocol was approved by the Ethics Committee of Shanghai Tenth People’s Hospital. Because data were collected retrospectively, informed consent on the use of coronary angiography was waived given the institutional ethics regulations with regard to observational study nature.

### 2.2 RA Procedure

Coronary angiography and PCI were performed using standard techniques. RA was performed using the Rotablator™ Rotational Atherectomy System, Boston Scientific, USA. The rotational speed was between 120,000 and 200,000 rpm, and the rotational speed was not allowed to drop more than 5000 rpm. The burr moved in a pecking motion with a single run time of less than 20 seconds. Verapamil, nitroglycerin, and heparin continuously were infused intracoronary during the RA procedure. After RA, the lesion is adequately dilated using cutting balloons or dilated balloons at the operator’s discretion. When adequate pre-treatment results were obtained, one or more stents were implanted. All patients were pretreated with a loading dose of dual antiplatelet therapy before procedures. Angio-IMR and angiography-derived fractional flow reserve (angio-FFR) were calculated from post-interventional coronary angiography images.

### 2.3 Angio-IMR and Angio-FFR Measurement

The angio-IMR measurement was conducted using the FlashAngio software (Rainmed Ltd., Suzhou, China). Details of the measurements and the angio-IMR procedures have been previously described [12]. Briefly, digital imaging and communications in medicine images (DICOM) coronary angiography images and the corresponding mean arterial pressure (MAP) were transferred to the FlashAngio workstation, and the interrogated vessels were selected for three-dimensional reconstruction. The estimated hyperemic Pa (${\mathrm{Pa}}_{\text{hyp}}$) was obtained by MAP, ${\mathrm{Pa}}_{\text{hyp}}$ = MAP × 0.2 when MAP ≥95 mmHg and MAP × 0.15 when MAP <95 mmHg [13]. The pressure drop (ΔP) from the inlet to the distal position of the vessel is obtained by computational pressure-flow dynamics using a validated method [13]. The distal pressure (${\mathrm{Pd}}_{\text{hyp}}$) of the vessel equals Pahyp minus ΔP. Thus, angio-FFR was calculated as follows:

(1) $$\text{ angio-FFR }=\frac{{\mathrm{Pd}}_{\mathrm{hyp}}}{{\mathrm{Pa}}_{\mathrm{hyp}}}$$

The thrombolysis in myocardial infarction frame count method [14] was used to calculate the mean flow velocity ($V_{\text{diastolic}}$) of blood flow passing through the selected length of the vessel (L). The angio-IMR was calculated as follows:

(2) $$\text{ angio-IMR }={\mathrm{Pd}}_{\text{hyp }}\left(\frac{L}{K\times V_{\text{diastole }}}\right)$$

**Fig. 2.**
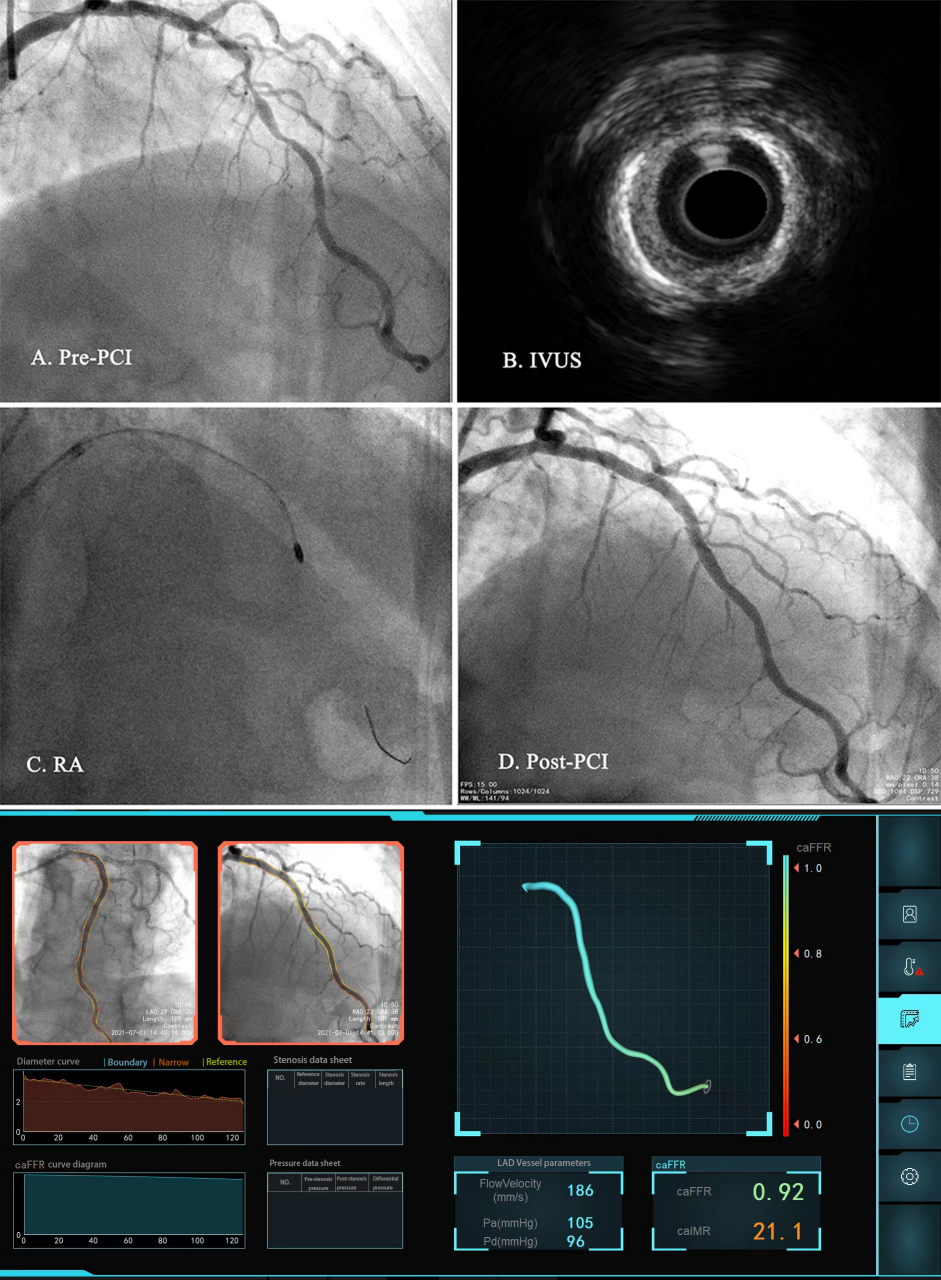
**Representative cases of coronary artery disease patients undergoing rotational atherectomy**. (A) Coronary angiography shows severe coronary stenosis and calcification in the LAD. (B) IVUS. (C) RA procedure. (D) Post-PCI. (E) Angio-IMR and angio-FFR calculation. Abbreviations: LAD, left anterior descending; IVUS, Intravascular ultrasound; RA, rotational atherectomy; PCI, percutaneous coronary intervention; angio-IMR, angiography-derived index of microcirculatory resistance; angio-FFR, angiography-derived fractional flow reserve.

K was a constant (K = 2.1) to adjust the difference between resting and hyperemic flow velocity [15]. The coronary angiograms were assessed by two trained cardiologists, and any disagreements were resolved by consensus. Fig. 2 shows a representative example of an angio-IMR analysis in patients undergoing the RA procedure.

### 2.4 Follow-up and Endpoint Definitions

Patients were followed for major adverse cardiovascular events (MACEs), including all-cause death, non-fatal myocardial infarction, and target vessel revascularization (TVR), by telephone interview or outpatient visit. All-cause death was defined as mortality from all causes. Non-fatal myocardial infarction was defined as ischemic symptoms and elevation of either troponin I >1.0 ng/mL or troponin T >0.1 ng/mL, regardless of the presence of ST-segment elevation or pathological Q waves in the electrocardiogram. TVR refers to a repeat procedure on the target vessel, either PCI or coronary artery bypass grafting. The median duration of follow-up was 21.7 (15.1–24.0) months. All interventional procedures were carried out by two interventional cardiologists in consultation.

### 2.5 Statistics Analysis

Continuous variables were expressed as mean ± standard deviation or median (interquartile range), as appropriate, and groups were compared using the *t*-test or Mann-Whitney U nonparametric test. Categorical variables were expressed as frequencies and percentages, and comparisons between groups were tested by chi-square analysis or Fisher exact test. CMD was defined as angio-IMR ≥25 [16]. The risk factors of CMD were analyzed by binary logistic regression. COX hazard models were used to assess predictors of MACEs and TVR, and variables included in the univariate analysis *p *< 0.1 were performed in a multivariate model. The cumulative incidence of MACEs, all-cause death, non-fatal myocardial infarction, TVR, and stroke from the index procedure to the most recent follow-up was expressed by Kaplan-Meier curves and compared using the log-rank test. Two sided *p* values < 0.05 were considered significant. All statistical analyses were conducted using SPSS version 22 software (IBM Inc, Armonk, NY, USA), and visualized by GraphPad software.8.0.1 (GraphPad Software Inc, San Diego, CA, USA).

## 3. Results

### 3.1 Patients and Lesion Characteristics according to the Angio-IMR

Clinical characteristics of the study population are presented in Table 1. A total of 118 patients with severe calcification undergoing RA were enrolled (72.39 ± 8.90 years, 68.9% males), of whom 40 patients (33.9%) had a previous PCI. The target vessel for RA was the left anterior descending coronary artery in 94 (79.7%) patients, the right coronary artery in 21 (17.8%) patients, and the left circumflex coronary artery in 3 (2.5%) patients, of whom 26 (22.5%) patients had coexisting left main disease. In this patient population, 112 had DES implantation after RA, with a mean number of stents implanted of 1.58 ± 0.80, and 6 patients had only percutaneous transluminal coronary angioplasty after RA. The frequency of RA was 2.65 ± 0.96, the duration of a single RA was 5.68 ± 1.71 sec, the mean angio-IMR after the RA procedure was 25.58 ± 7.93, and the mean angio-FFR was 0.92 ± 0.03.

**Table 1.** **General characteristics of the study population and lesion sets**.

Variables			Overall	Angio-IMR ≥25	Angio-IMR <25	*p*-value
			(n = 118)	(n = 54)	(n = 64)	
Demographics						
	Age, years		72.39 ± 8.90	71.22 ± 9.31	73.22 ± 8.53	0.227
	Male		82 (68.9)	42 (77.8)	40 (62.5)	0.073
Cardiovascular risk factors						
	Hypertension		93 (78.8)	40 (74.1)	53 (82.8)	0.247
	Diabetes mellitus		58 (49.2)	27 (50.0)	31 (48.4)	0.866
	Hyperlipidemia		13 (11.0)	7 (13.0)	6 (9.4)	0.535
	Smoking		30 (25.4)	12 (22.2)	18 (28.1)	0.463
	Stroke		26 (22.0)	9 (16.7)	17 (26.6)	0.196
	Prior PCI		40 (33.9)	16 (29.6)	24 (37.5)	0.368
	LVEF, %		57.98 ± 8.81	58.70 ± 8.62	57.45 ± 8.94	0.443
Medication use						
	Asprin		113 (95.8)	52 (96.3)	61 (95.3)	0.792
	P2Y12 inhibitor		117 (99.2)	53 (98.1)	64 (100)	0.274
	Statin		115 (97.5)	51 (94.4)	64 (100)	0.056
	Beta-blocker		76 (64.4)	37 (68.5)	39 (60.9)	0.392
	ACEI/ARB		83 (70.3)	35 (64.8)	48 (75.0)	0.228
Coronary angiography						
	Target vessel					
	Left main disease		26 (22.5)	9 (16.7)	17 (26.6)	0.196
		RCA	21 (17.8)	14 (25.9)	7 (10.9)	
		LAD	94 (79.7)	39 (72.2)	51 (85.9)	
		LCX	3 (2.5)	1 (1.9)	2 (3.1)	
	Diameter stenosis		86.86 ± 7.84	87.93 ± 7.56	85.95 ± 8.02	0.174
	TIMI grade					
		0	5 (4.2)	4 (2.3)	1 (1.6)	0.243
		1	3 (2.5)	2 (3.7)	1 (1.6)	
		2	8 (6.8)	2 (3.7)	6 (9.4)	
		3	102 (86.4)	46 (85.2)	56 (87.5)	
	Number of vessels					
		1	18 (15.3)	9 (16.7)	9 (14.1)	0.085
		2	40 (33.9)	17 (31.5)	23 (35.9)	
		3	60 (50.8)	28 (51.9)	32 (50.0)	
IVUS (n = 101)						
	MLA, ${\mathrm{mm}}^{2}$		3.00 ± 1.16	2.96 ± 1.35	3.01 ± 0.97	0.826
	Calcification arc		323.50 ± 48.70	321.67 ± 54.18	325.63 ± 43.71	0.661
	Plaque load, %		80.99 ± 7.23	81.09 ± 8.58	80.89 ± 5.90	0.884
RA						
	Rotation speed		161.7 ± 8.2	161.39 ± 7.00	162.03 ± 9.12	0.673
	Duration of single RA, s		5.68 ± 1.71	5.41 ± 1.21	5.91 ± 2.21	0.102
	Frequency of RA, times		2.65 ± 0.96	2.61 ± 0.70	2.7 ± 1.21	0.599
	Maximum burr size, mm					
		1.25	20 (16.9)	7 (13.0)	13 (20.3)	0.241
		1.50	97 (82.2)	46 (85.2)	51 (79.7)	
		1.75	1 (0.8)	1 (1.9)	0 (0)	
	Cutting balloon		9 (7.6)	3 (5.6)	6 (9.4)	0.506
	Number of stents		1.58 ± 0.80	1.48 ± 0.82	1.67 ± 0.78	0.198
	Angio-FFR		0.92 ± 0.03	0.94 ± 0.02	0.90 ± 0.03	<0.001
	Angio-IMR		25.58 ± 7.93	32.45 ± 6.08	19.71 ± 3.09	<0.001

Values are mean ± SD or n (%). Abbreviations: PCI, percutaneous coronary intervention; LVEF, left ventricular ejection fraction; ACEI, angiotensin-converting enzyme inhibitor; ARB, angiotensin II receptor blocker; RCA, right coronary artery; LAD, left anterior descending; LCX, left circumflex; TIMI, thrombolysis in myocardial infarction; IVUS, Intravascular ultrasound; RA, rotational atherectomy; Angio-IMR, coronary angiography-derived index of microcirculatory resistance; Angio-FFR, coronary angiography-derived fractional flow reserve; MLA, minimum luminal area.

Fifty-four patients (45.8%) exhibited CMD by angio-IMR ≥25, and the mean angio-IMR (32.45 ± 6.08 vs. 19.71 ± 3.09, *p *< 0.001) and angio-FFR (0.94 ± 0.02 vs. 0.90 ± 0.03, *p *< 0.001) were significantly higher in the group with angio-IMR ≥25 than the group with angio-IMR <25. There were no statistical differences between the two groups in terms of cardiovascular risk factors, medication use, and other coronary physiological indices (*p *> 0.05).

### 3.2 Predictors of CMD

**Supplementary Table 1** shows the univariable and multivariable binary logistic regression analyses used to predict CMD. By univariable logistic regression analysis, variables with* p <* 0.2 were examined in a multivariable model, the results showed that angio-FFR was independently associated with CMD (*p *< 0.001).

### 3.3 Prognostic Implication of Angio-IMR

**Fig. 3.**
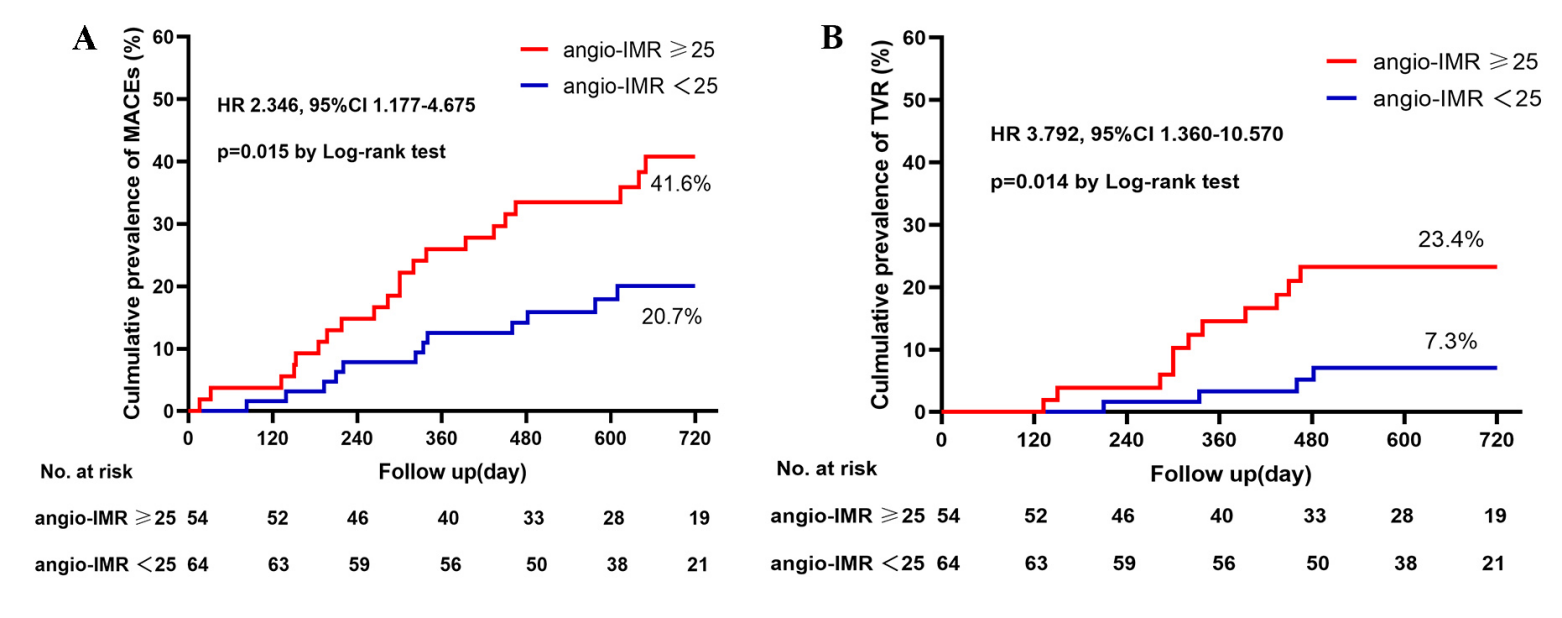
**Cumulative prevalence of MACEs (A) and TVR (B) in patients with rotational atherectomy stratified by angio-IMR**. MACE is a composite of cardiac death, non-fatal myocardial infarction, TVR, and stroke. Abbreviations: MACEs, major adverse cardiovascular event; TVR, target vessel revascularization; Angio-IMR, coronary angiography-derived index of microcirculatory resistance; HR, hazard ratio.

The median duration of follow-up was 21.7 months (IQR: 16.1–24.0 months), and completed follow-up was obtained in all 118 patients. The follow-up results are presented in **Supplementary Table 2**. There were 33 MACEs, including 11 all-cause deaths, seven cardiac deaths, six non-fatal myocardial infarctions, 15 TVRs, and one stroke. In the Kaplan-Meier analysis, patients with angio-IMR ≥25 had a significantly higher prevalence of MACEs (41.6% vs. 20.7%; hazard ratio: 2.346; 95% confidence interval: 1.177–4.675; Log-rank *p* = 0.015) and TVR (23.4% vs. 7.3%; hazard ratio: 3.792; 95% confidence interval: 1.360–10.570; Log-rank *p* = 0.014) were significantly higher than in patients with angio-IMR <25 (Fig. 3), whereas the cumulative hazard of all-cause death, cardiac death, non-fatal myocardial infarction, and stroke was similar between the two groups (Log-rank *p *> 0.05) (**Supplementary Table 2**).

In the univariate and multivariate COX regression analyses for predictors of MACEs, cardiovascular risk factors (sex, age, hypertension, diabetes mellitus, hyperlipidemia, smoking, previous PCI), coronary physiological indices (diameter of stenosis, left main stem lesion, triple vessel lesion), and coronary procedural parameters (angle of calcification, MLA, plaque load, single spin duration, and number) were first studied by univariate analysis. The variables with *p *< 0.1 were then analyzed by multivariate COX regression. The results suggested that angio-IMR ≥25 and decreased left ventricular ejection fraction were independent predictors of MACEs (Table 2). Similarly, univariate and multivariate COX regression analyses were performed for TVR, and the results demonstrated that angio-IMR ≥25 and reduced MLA independently predicted TVR (Table 3).

**Table 2.** **Independent predictors of MACEs**.

Variables	Univariate			Multivariate		
	*p*-value	HR	95% CI	*p*-value	HR	95% CI
Male	0.099	2.003	0.887–4.573			
LVEF, %	0.041	0.969	0.940–0.999	0.039	0.969	0.941–0.998
Triple-vessel disease	0.023	2.232	1.116–4.456			
Diameter stenosis, %	0.050	1.049	1.000–1.100			
Plaque load, %	0.051	1.047	1.000–1.096			
MLA, ${\mathrm{mm}}^{2}$	0.040	0.739	0.554–0.986			
Time of RA, s	0.061	0.561	0.307–1.027			
Frequency of RA, times	0.077	0.610	0.353–1.056			
Angio-IMR ≥25	0.010	2.450	1.240–4.839	0.015	2.367	1.185–4.729

Abbreviations: HR, hazard ratio; CI, confidence interval; MACEs, major adverse cardiovascular events; RA, rotational atherectomy; LVEF, left ventricular ejection fraction; Angio-IMR, coronary angiography-derived index of microcirculatory resistance; MLA, Minimum luminal area.

**Table 3.** **Independent predictors of TVR**.

Variables	Univariate			Multivariate		
	*p*-value	HR	95% CI	*p*-value	HR	95% CI
Hypertension	0.098	0.432	0.160–1.168			
Angio-IMR ≥25	0.022	3.373	1.188–9.580	0.035	3.166	1.083–9.255
Calcification arc	0.073	1.012	0.999–1.026			
Plaque load, %	0.077	1.062	0.993–1.136			
MLA, ${\mathrm{mm}}^{2}$	0.006	0.565	0.376–0.849	0.013	0.421	0.213–0.831

Abbreviations: HR, hazard ratio; CI, confidence interval; TVR, target vessel revascularization; Angio-IMR, coronary angiography-derived index of microcirculatory resistance; MLA, Minimum luminal area.

## 4. Discussion

To the best of our knowledge, the present study is the first to focus on the microcirculatory characteristics and prognostic implications after RA in patients with CAD according to angio-IMR. The main findings of the current study are: (1) The incidence of CMD in patients with CAD assessed by angio-IMR measured immediately after the RA procedure was 45.8%. (2) Angio-FFR was independently associated with the occurrence of CMD. (3) CAD patients undergoing RA with angio-IMR ≥25 had a greater risk of MACEs and TVR than patients with preserved angio-IMR.

### 4.1 Assessment of Microvascular Function with Angio-IMR 

Coronary revascularization can restore epicardial vessel blood flow perfusion, but this may not be equivalent to increased myocardial blood flow perfusion. Myocardial perfusion is supplied by the epicardial macrovascular system in addition to the much larger microvascular system. It is well known that patients with a combined CMD have a worse prognosis [5]. There are several methods for evaluating the microcirculatory system, and noninvasive assessment techniques such as positron emission tomography and IMR are underutilized. In contrast, among the invasive assessment tools, wire-based IMR and coronary flow reserve (CFR) are the most reliable indicators for evaluating microcirculation [9], with CFR being relatively affected by hemodynamic and heart rate changes, while IMR is highly reproducible and specific for the microcirculation [17, 18]. Previous studies have shown that microcirculatory dysfunction assessed by IMR is independently associated with adverse outcomes in various cardiovascular diseases [19, 20, 21, 22]. However, the invasiveness of physiological assessments limits their application. The angio-IMR, a reliable alternative to wire-derived IMR, was developed based on the principles of computational flow and pressure dynamics, and has greatly simplified the assessment of IMR. Angio-IMR not only showed a high correlation with wire-derived IMR but also have prognostic implications [23, 24].

### 4.2 RA Treats Severe Coronary Artery Calcification

Studies have shown that 30.8% of patients with elective DES implantation have moderate to severe coronary calcification and that severe calcification is associated with an increased incidence of MACE resulting in adverse patient prognosis [25]. Severe coronary artery calcification can compromise coronary interventions by increasing the incidence of stent delivery failure, impairing stent expansion, and even causing dilatation balloon rupture [26]. More tools are now available to tackle severe coronary artery calcification, such as cutting balloons, intravascular lithotripsy, orbital atherectomy, and laser, but RA remains the predominant technique to treat calcified plaques [27, 28]. The plaque modification role of RA can effectively reduce calcification and fibrous plaque volume, broaden the lumen, and enhance stent apposition [4, 29, 30]. In addition, plaque modification by RA has been shown to lower the incidence of stent restenosis and malposition [31, 32]. Nevertheless, TVR and target vessel failure (TVF) rates remain high in patients with severe coronary calcium lesions; hence, the need to explore the association between RA procedures for severe coronary calcification and microvascular function.

### 4.3 Incidence and Predictor of CMD in RA Patients

In our study, the incidence of CMD after RA was 45.8%, traditional cardiovascular risk factors are independent of CMD. In a 120-patient study of CFR after chronic total occlusions (CTO), CMD was diagnosed in 46% of patients [33]; A multicenter, prospective study by Kobayashi *et al*. [34] reported that 59.1% of patients with suspected myocardial ischemia had no CMD and 40.9% of patients had CMD in single or multiple vessels. Univariate and multivariate logistic regression analysis showed that clinical factors and coronary severity did not predict CMD [34]. This is similar to our findings in that the incidence of CMD in patients with severe coronary artery calcification assessed immediately after RA was consistent with that in patients with CTO and slightly higher than that in patients with stable suspected ischemia, and that clinical factors and RA procedural parameters were not predictive of CMD. However, the angio-FFR was independently associated with the occurrence of CMD, an increased angio-FFR increased the risk of CMD, which suggested a link between coronary blood flow and microvascular function after RA. The exact mechanism of this is currently unclear and needs to be confirmed by further studies.

### 4.4 Increased Angio-IMR Predicts MACEs

The prognostic significance of IMR after PCI for stable CAD has been demonstrated [19]. Recent studies have shown that angio-IMR can predict the outcome of patients with acute myocardial infarction and after PCI [24, 35]. In the present study, we found significantly higher MACEs in patients with angio-IMR ≥25, with a cumulative MACEs incidence of 41.6% compared with 20.7% for no CMD patients and an overall MACEs rate of 30.6%, in which all-cause mortality was 12.5%, cardiac death 8.0%, non-fatal myocardial infarction and TVR 6.4% and 14.5%, respectively. A Korean registry study showed a similar incidence of target vessel failure at 18-month follow-up after RA in CTO and non-CTO patients, 14.1% and 16.7%, respectively [36]. In contrast, in a study of stable CAD and acute coronary syndrome with RA, the incidence of MACEs was 39.9% and 22.4%, respectively, at 24-month follow-up [37]. Two-year clinical outcomes of RA for severely calcified lesions after next-generation DES implantation showed MACEs (cardiac death, myocardial infarction, clinically driven target lesion revascularization, and definite stent thrombosis) of 20.3% (7.0%, 2.1%, 18.1%, and 2.1%, respectively) [38]. Another DES study of 188 European patients with RA after a median follow-up of 15 months, showed a cumulative MACEs incidence of 17.7% and TVR of 9.9% [39]. In a study of RA for in-stent restenosis, the 12-month follow-up results showed target lesion revascularization rates of 40.7%, 35.0%, and 27.3% after balloon angioplasty, DES implantation, and drug-coated balloon angioplasty, respectively [40]. This suggests that in patients after RA procedures, the incidence of MACEs remains high, especially with TVR.

In our study, angio-IMR ≥25 was an independent predictor of MACEs and TVR. A study addressing operative variables showed that in a multivariate analysis affecting prognosis, patients with a single burr had a better prognosis than those with a non-single burr, independent of burr size and location [41]. In contrast, single burr patients accounted for 90% of the patients in this study. This may explain the increased occurrence of high MACEs and TVR in patients with CMD after RA.

This study has important predictive implications for patients with severely calcified coronary artery lesions. Interventionalists now have more tools to deal with various complicated coronary lesions. However, while anatomic obstruction is relieved, the recovery of CMD is essential. Angio-IMR is a simple and convenient index to assess the state of microcirculation after RA, and we have found that elevated angio-IMR increases the risk of MACEs; therefore, we can use the strategies guided by angio-IMR to improve the prognosis of patients with microcirculatory dysfunction. Studies have shown that statins, angiotension converting enzyme inhibitors, beta-blockers and anti-anginal treatments such as nicorandil and trimetazidine help to improve the prognosis of patients with CMD [9]. However, to date, there are no specific treatment strategies for CMD that have been validated in scale randomised clinical trials, and therefore treatment of patients with CMD should be targeted at risk factors. We will also further explore the role of angio-IMR in guiding the treatment of microcirculatory dysfunction in the future.

### 4.5 Study Limitation

This study has several study limitations. First, the study’s sample size was small, it was a retrospective study, and the findings need to be confirmed by a prospective study with a larger sample size. Second, we only analyzed the target vessels in which the RA procedure was performed and did not include reference vessels for comparison. Third, the study used angio-IMR to assess microcirculatory function, and the analysis results were affected by the contrast images. Thus, five patients (4.2%) were excluded because the images did not meet the requirements.

## 5. Conclusions

The incidence of CMD in patients with severe coronary calcification after the RA procedure was 45.8%. Angio-IMR ≥25 was an independent and significant predictor of MACEs and TVR in patients after the RA procedure.

## Data Availability

The data that support the findings of this study are available from Shanghai Tenth People’s Hospital Medical Record System but restrictions apply to the availability of these data, which were used under license for the current study, and so are not publicly available.

## Abbreviations

RA, rotational atherectomy; CMD, coronary microvascular dysfunction; Angio-IMR, angiography-derived index of microcirculatory resistance; CAD, coronary artery disease; MACEs, major adverse cardiovascular events; TVR, target vessel revascularization; PCI, percutaneous coronary intervention; DES, drug-eluting stent; MAP, mean arterial pressure; MLA, minimum luminal area.

## Supplementary Material

Supplementary material associated with this article can be found, in the online version, at https://doi.org/10.31083/j.rcm2405131.
