# Supplementary material for: Prognostic Value of Angiography-Derived Index of Microcirculatory Resistance in Patients with Coronary Artery Disease Undergoing Rotational Atherectomy
Source: Rev Cardiovasc Med. 2023 Apr 27;24(5):131. doi: 10.31083/j.rcm2405131 (PMC11273008; doi:10.31083/j.rcm2405131)
Supplement: Supplementary file 1 [file 2153-8174-24-5-131-s1.zip › 2153-8174-24-5-131-s1.docx]

*Supplementary materials*

Supplementary Table1. Associations of CMD with conventional cardiovascular risk factors and parameters of coronary artery lesions and procedures.

| Variables | Univariate | | | Multivariate | | |
| --- | --- | --- | --- | --- | --- | --- |
|  | *p*-value | OR | 95% CI | *p*-value | OR | 95% CI |
| Male  Age  Hypertension  Diabetes mellitus  Hyperlipidemia  Stroke  Smoking  Prior PCI  LVEF  Left main disease  Triple vessel lesion  Number of stents  Diameter stenosis  Calcification arc  Plaque load  MLA  Time of single RA  Frequency of RA  Blur size  Angio-FFR, per 0.01u increase | 0.075  0.226  0.250  0.866  0.537  0.200  0.464  0.369  0.442  0.200  0.841  0.198  0.175  0.658  0.879  0.820  0.127  0.599  0.207  <0.001 | 2.100  0.975  0.593  1.065  1.440  0.553  0.730  0.702  1.017  0.553  1.077  0.736  1.034  0.998  1.004  0.964  0.825  1.109  12.291  2.247 | 0.928-4.754  0.935-1.016  0.244-1.444  0.516-2.197  0.453-4.576  0.224-1.367  0.315-1.694  0.324-1.520  0.974-1.062  0.224-1.367  0.522-2.223  0.462-1.174  0.985-1.084  0.991-1.006  0.955-1.056  0.704-1.320  0.644-1.056  0.755-1.629  0.250-603.355  1.647-3.066 | 0.388  0.144  0.317  0.940  0.227  0.416  <0.001 | 1.595  0.390  0.549  0.978  1.045  0.880  2.247 | 0.552-4.607  0.111-1.379  0.170-1.775  0.542-1.764  0.973-1.121  0.646-1.198  1.618-3.119 |

Abbreviations: CMD, coronary microvascular dysfunction; OR, odds ratio; CI, confidence interval; PCI, percutaneous coronary intervention; LVEF, left ventricular ejection fraction; RA, rotational atherectomy; MLA, minimum luminal area; Angio-FFR, coronary angiography-derived fractional flow reserve.

Supplementary Table 2. Clinical outcomes in patients with or without CMD.

| Outcomes | Overall | Angio-IMR≥25  (n = 54) | Angio-IMR<25  (n = 64) | | P-value |
| --- | --- | --- | --- | --- | --- |
| MACEs  All death | 33(30.6)  11(12.5) | 21(41.6)  5(13.4) | 12(20.7)  6(11.5) | 0.015  0.880 | |
| Cardiac death | 7(8.0) | 3(7.7) | 4(8.1) | 0.975 | |
| NFMI | 6(6.4) | 4(10.1) | 2(3.3) | 0.247 | |
| Stroke | 1(0.9) | 1(2.0) | 0(0.0) | 0.265 | |
| TVR | 15(14.5) | 11(23.4) | 4(7.3) | 0.014 | |

Values are n (%). Abbreviations: CMD, coronary microvascular dysfunction; MACEs, major adverse cardiovascular events; NFMI, non-fatal myocardial infarction; TVR, target vessel revascularization. P-values were derived using the log-rank test.
